# Utilization of lithium incorporated mesoporous silica for preventing necrosis and increase apoptosis in different cancer cells

**DOI:** 10.1186/s13065-019-0535-5

**Published:** 2019-01-30

**Authors:** Kamel A. Saleh, Sharah A. A. Aldulmani, Nasser S. Awwad, Hala A. Ibrahium, Tahani H. Asiri, Mohamed S. Hamdy

**Affiliations:** 1 Department of Chemistry, Faculty of Science, King Khalid University, P.O. Box 9004, Abha, Saudi Arabia; 2 Department of Biology, Faculty of Science, King Khalid University, P.O. Box 9004, Abha, Saudi Arabia; 3 Department of Biology, Nuclear Materials Authority, P.O. Box 530, El Maadi, Egypt

**Keywords:** Lithium silicate, Mesoporous silica, Necrosis, Li-TUD-1, NA-K pump, Apoptosis

## Abstract

There are many molecules used as a drug carrier. TUD-1 is a newly synthesized mesoporous silica (SM) molecule possess two important features; consists of mesoporous so it is very suitable to be drug carrier in addition to that it has the ability to induce apoptosis in cancer cells. However, the effect of TUD-1 appears to act as cell death inducer, regardless of whether it is necrosis or apoptosis. Unfortunately, recent studies indicate that a proportion of cells undergo necrosis rather than apoptosis, which limits the use of TUD-1 as a secure treatment. On the other hand, lithium considered as necrosis inhibitor element. Hence, the current study based on the idea of producing a new Li-TUD-1 by incorporated mesoporous silica (TUD-1 type) with lithium in order to produce a new compound that has the ability to activate apoptosis by mesoporous silica (TUD-1 type) and at the same time can inhibit the activity of necrosis by lithium. Herein, lithium incorporated in TUD-1 mesoporous silica by using sol–gel technique in one-step synthesis procedure. Moreover, lithium incorporated in TUD-1 with different loading in order to form different active sites such as isolated lithium ions, nanoparticles of Li_2_O, and bulky crystals of Li_2_O. The ability of the new compounds to induce apoptosis and prevent necrosis was evaluated on three different types of cancer cell lines, which are; liver HepG-2, breast MCF-7, and colon HCT116. The obtained results show that Li-TUD-1 has the ability to control necrosis and thus reduce the side effects of treatments containing silica in the case of lithium added to them, especially in chronic cases. This opinion has demonstrated by the significant increase in the IC_50_ value and cell viability compared to control groups. Consequently, the idea is new, so it needs more develop and test with materials that have a more apoptotic impact than silica to induce apoptosis without induction of necrosis.

## Introduction

The invention of a new anti-cancer treatment is related to understanding the mechanism of entering the molecules into the cell and then following the pathway(s) which the treatment will activate to induce cells to apoptosis. Hence, molecules with high permeability or loading high-impact molecules should be selected for vectors that have the ability to enter cells easily [1, 2]. But not every molecule that can enter cells has the ability to activate the appropriate mechanisms to kill or to keep cancer cells under control. For example, mesoporous silica nanoparticles (MSN) have the ability to enter the cell by enhancing permeability or retention effect [3]. These particles have gained their importance from possessing a large surface area and their pores volume [4–6].

Consequently, many vital applications that rely directly on these properties have emerged like immediate/sustained drug delivery systems, bio-therapeutic agent delivery, controlled/targeted drug delivery systems [6–9]. Moreover, MSN has special role in bio-imaging applications and bioactive materials for tissue regeneration [10–12]. In addition to that, it can be considered as biocompatible materials [13]. However, the main challenge lies in the ability of silica not to induce apoptosis but its ability to activate necrosis mechanisms. While the ideal anti-cancer therapies and drug design seek to induce apoptosis without activating necrosis. The reason for that, cells undergoing apoptosis will produce apoptotic bodies and membrane blebs which activate the immune system [14–16]. Wherefore, mesoporous silica alone is not favored as anti-cancer treatment despite its ability to penetrate and eliminate as many cancer cells as possible, due to its ability to induce necrosis along with apoptosis. In order to fix this problem anew modified mesoporous silica molecule should be designed.

On the other hand, lithium has been tested for many years as a treatment for the mood disorders [17, 18]; it can be deemed a safe treatment for normal cells because it protects nerve cells from necrosis by inhibiting the activity of anti-apoptosis bcl-2 gene [19–22]. In addition, to modulate cytokine production, gene expression lithium can induce apoptosis and stimulates the cell division of neuroblasts in primary cultures [22, 23]. Based on the above, it can be said that silica has a great role as a drug vector and at the same time apoptosis promoter, but its side effect is to stimulate necrosis. Whereas lithium stimulates the cell to divide, if it fails, it prevents it from going to necrosis by inhibition of bcl-2.

In an attempt to produce a double-sided effect and triple feature drug, lithium was incorporated onto mesoporous silica TUD-1 in order to combine the three good features (enhances apoptosis by silica, decreases necrosis by lithium, in addition, to use as drug delivery molecule) this study was designed. The new Li-TUD-1 that combines the qualities of silica in terms of easy entry to the cell as a carrier and urged by the cell to apoptosis, and lithium, which protects the cell from going to necrosis. This new Li-TUD-1 will be known as Li-TUD-1.

## Materials and methods

### Prepared of Li incorporated silica (Li-TUD-1)

Four Li-TUD-1 samples were prepared according to a molar ratio composition of SiO_2_ : xLi : 0.5TEAOH : 1TEA : 11H_2_O, where x = 0.01, 0.05, 0.1, and 0.2. The prepared materials were synthesized by mixing a solution consisting of triethanolamine (TEA, 97%, ACROS) and LiNO_3_ (99% Sigma) with tetraethyl orthosilicate (TEOS, +98%, ACROS) under vigorous stirring. After 30 min, tetraethyl ammonium hydroxide (TEAOH, 35%, Aldrich) was added dropwise and the overall mixture stirred for at least 2 h at room temperature. The resulting homogeneous solution/gel was aged at room temperature for 24 h and then dried at 371 K for another 24 h. The obtained solid was ground and hydrothermally treated in a 50 mL Teflon-lined stainless steel autoclave at 451 K under autogenous pressure for 4 h. The obtained solid was ground again and then calcined in static air at 873 K for 10 h applying a heating ramp rate of 1 K/min. The obtained solids were stored in a clean glass bottles and kept in dissector. The four samples were coded as Li-x, where x is the loading wt% which are 1, 5, 10 and 20.

### Characterizations

The prepared materials were characterized by several techniques to understand the structure of each sample. X-ray diffraction (XRD) measurements were carried out with Shimadzu LabX-XRD-6000 diffractometer with *CuK*_*α*_ (*λ* = 1.5406 Å) radiation and secondary monochromator attached with Shimadzu software with pdf-2 library for the analysis of XRD data. The data collection was carried out under ambient conditions. Moreover, FT-IR spectra of Li-TUD-1 were recorded using THERMO SCIENTIFIC, DXR FT-IR spectrometer by KBr pellet method in the wavenumber range of 4000–400 cm^−1^. Finally, the morphological structure of prepared materials were characterized by field emission scanning electron microscope (FE-SEM) (JSM-7500 F; JEOL-Japan) equipped with energy dispersive spectroscopy (EDS) microanalysis system.

### Antitumor activity study

All chemicals used in this study (SulphoRhodamine-B (SRB, Tris-HCl, Tricloroacitic acid (TCA)) were purchased from Sigma Chemical Co. (St. Louis, MO, USA). While media and related products were supplemented from Gibco/Life Technologies Co (Carlsbad, CA, USA). Cells Human hepatic carcinoma (HEPG-2), breast (MCF-7) and colon (HCT116) used in the current study were purchased from Vacsera (Giza, Egypt). In preparation to treatment, cells maintained in cell culture media RPMI which is supplemented with 100 µg/mL streptomycin/penicillin and 10% heat-inactivated (FBS) fetal bovine serum. The cytotoxicity of the elements and were tested against HEPG-2, MCF-7, and colon HCT116 tumor cell lines. Confluent 80% cells were exposed to the Li alone, TUD-1 alone and Li-TUD-1 with (0.01, 0.1, 1, 10, 100 µg/mL) for 72 h. SRB evaluation test used to determine the IC_50_ value (Table 1). This value is beneficial and important to evaluate the effective dose for each concentration. The data were analyzed using Sigma Plot version 12.0.

**Table 1:** The impact of Li, TUD-1, and Li-TUD-1 on cell viability against cancer cell lines

Cell type	Cell viability					
	TUD-1 (%)	Li (%)	Li-TUD-1			
			1	5	10	20
HEPG2	53	87.9	84.2 ± 1.97	89.5 ± 2.40	93.42 ± 1.44	92.8 ± 1.11
MCF-7	56	61.4	75.9 ± 1.54	77.91 ± 3.37	80.5 ± 3.9	77.6 ± 1.75
HCT116	45	90.5	90.13 ± 1.76	92.15 ± 2.46	94.04 ± 2.14	92.42 ± 1.71

## Results and discussion

### Characterization data

**Fig. 1:**
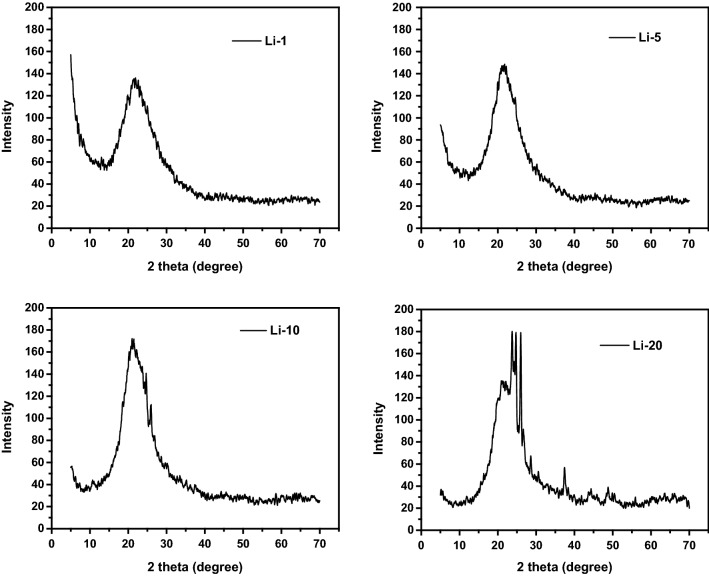
The X-ray diffraction (XRD) patterns of the prepared Li-TUD-1 samples

XRD spectra of Li-TUD-1 samples were measured within the 2*θ* range of 5–70^ο^ are presented in Fig. 1. The XRD patterns for the entire samples showed one broad intensive peak at 23^ο^ which can be attributed to the amorphous phase of SiO_2_ [24]. Moreover, no more phases were observed in Li-1 sample as an indication for the total incorporation of Li ions in the silica framework. As the loading of Li increased, new peaks were observed at 23.7^ο^, 24.6^ο^, 26.0^ο^, 26.6^ο^, 28.5^ο^, 37.7^ο^ 2that. These peaks can be attributed to the formation of crystalline phases of Li_4_SiO_4_ and Li_8_SiO_6_. The crystals formation of lithium silicate different phases was also observed by Janes and co-working during the synthesis of Li-SBA-15 [25]. Therefore; the XRD results showed the formation of isolated Li sites in Li-1, nanoparticles of crystalline Li-silicate phases in Li-5 and Li-10 samples and finally, bulky extra-framework crystals of Li-silicate different phases were detected. The formation of different dopant structure by increasing the loading of the dopant also observed in the work of Hamdy and coworkers [26, 27].

**Fig. 2:**
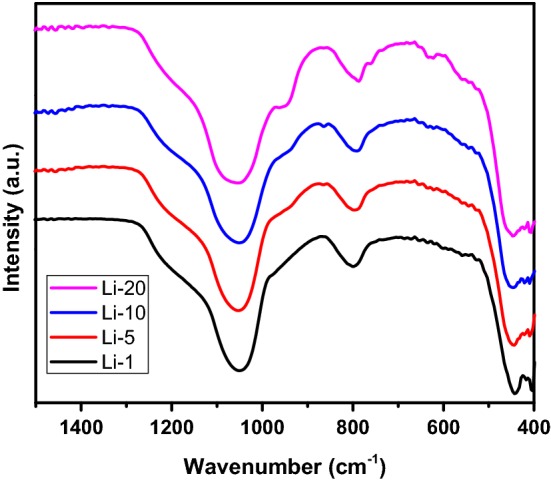
The FTIR spectra of the prepared Li-TUD-1 samples

FT-IR spectra of Li-TUD-1 samples collected at ambient conditions without further treatment (Fig. 2). All the peaks are dominated with two peaks at 1054 and 804 cm^−1^, which can be attributed to the asymmetric stretching and symmetric modes of Si–O–Si lattice vibrations, respectively [28, 29]. It established that the peak near 950 cm^−1^ could be attributed to the surface silanol group (Si–OH). The intensity of this peak was stronger with Li content; this can be explained by the formation of more Si–OH groups due to the hydrophilicity of Li-silicate crystals, which adsorb more water molecules. These adsorbed water molecules interact with the Si–O–Si of TUD-1 to form the surface hydroxyls. Hence, the intensity of the peak became stronger [30]. Moreover, the intensity of 804 cm^−1^ band was gradually decreasing with Li content, and other bands at 779, 740, and 628 cm^−1^ were clearly seen. These bands can be attributed to the formation of lithium silicate crystals. Finally, the obtained FT-IR results are in a good agreement with the XRD results.

**Fig. 3:**
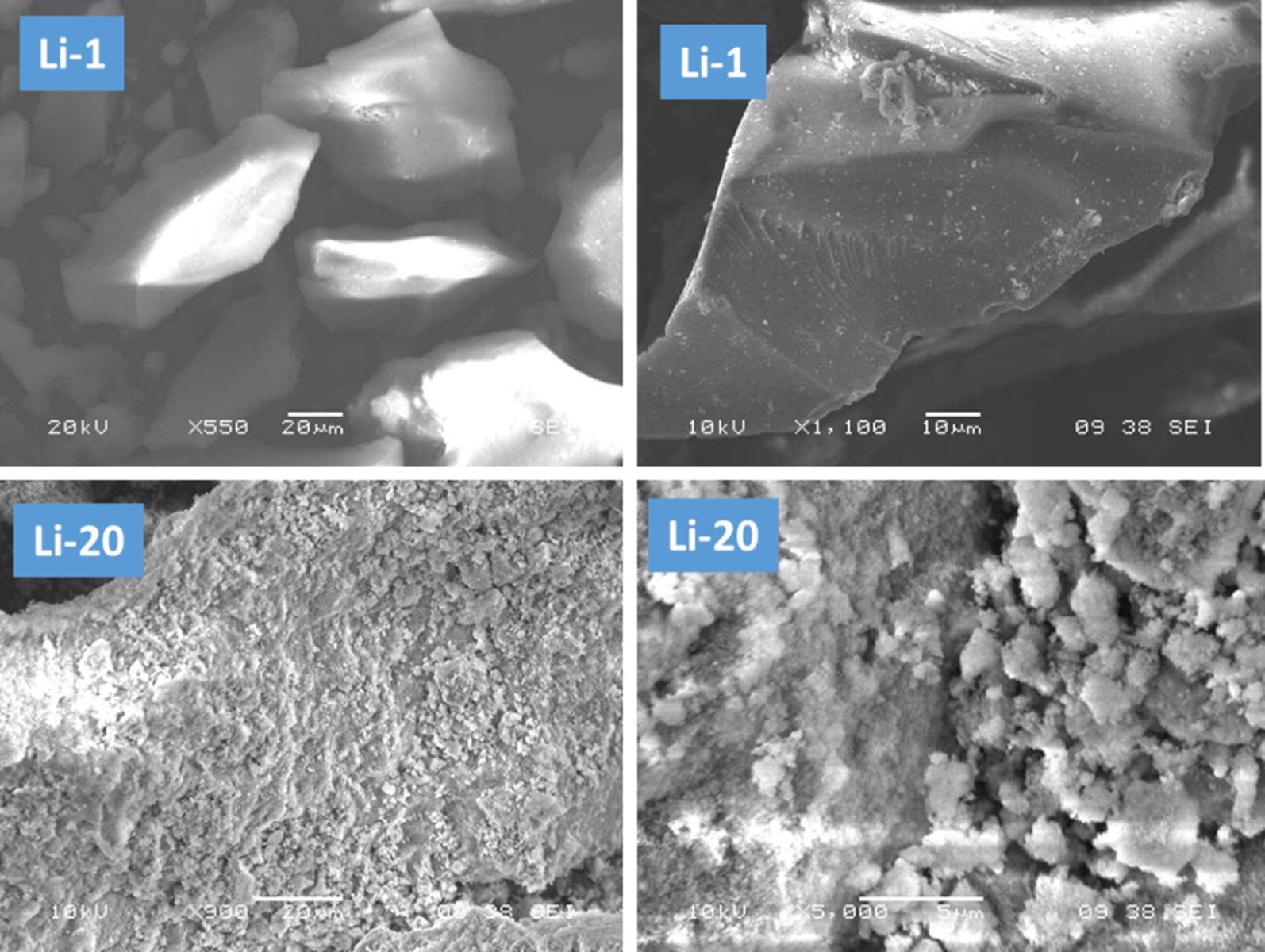
SEM micrographs of the prepared Li-1 and Li-20 samples

Figure 3 shows the scanning electron microscope (SEM) micrographs of Li-1 and Li-20 samples. In Li-1 micrographs, only irregular smooth surface particles were detected. The surface morphological structure of Li-1 sample is typical to that of TUD-1 silica material [31]. No extra-framework particles could be detected. However, in Li-20 sample, the micrographs show in addition to the silica particles, extra-framework of small spherical lithium silicate particles. This result is in-line with XRD and FT-IR results, which all are indicated that at high loading of Li, extra-framework of lithium silicate phase are formed.

### The antitumor behavior of Li-TUD-1 samples

The obtained results confirmed that the mesoporous silica (TUD-1), Li, and the Li-TUD-1 exhibit different cytotoxicity impact against HEPG-2, MCF-7 and colon HCT116 tumor cell lines with the IC_50_ values >100 µg/mL, for TUD-1 and Li separately while the impact was < 1000 in all cell lines treated with Li-TUD-1 for each concentration (0.01, 0.1, 1, 10, 100 µg/mL). These results clearly prove the synergy between Li and silica in the increasing IC_50_ values to be more effective in different cell lines.

**Fig. 4:**
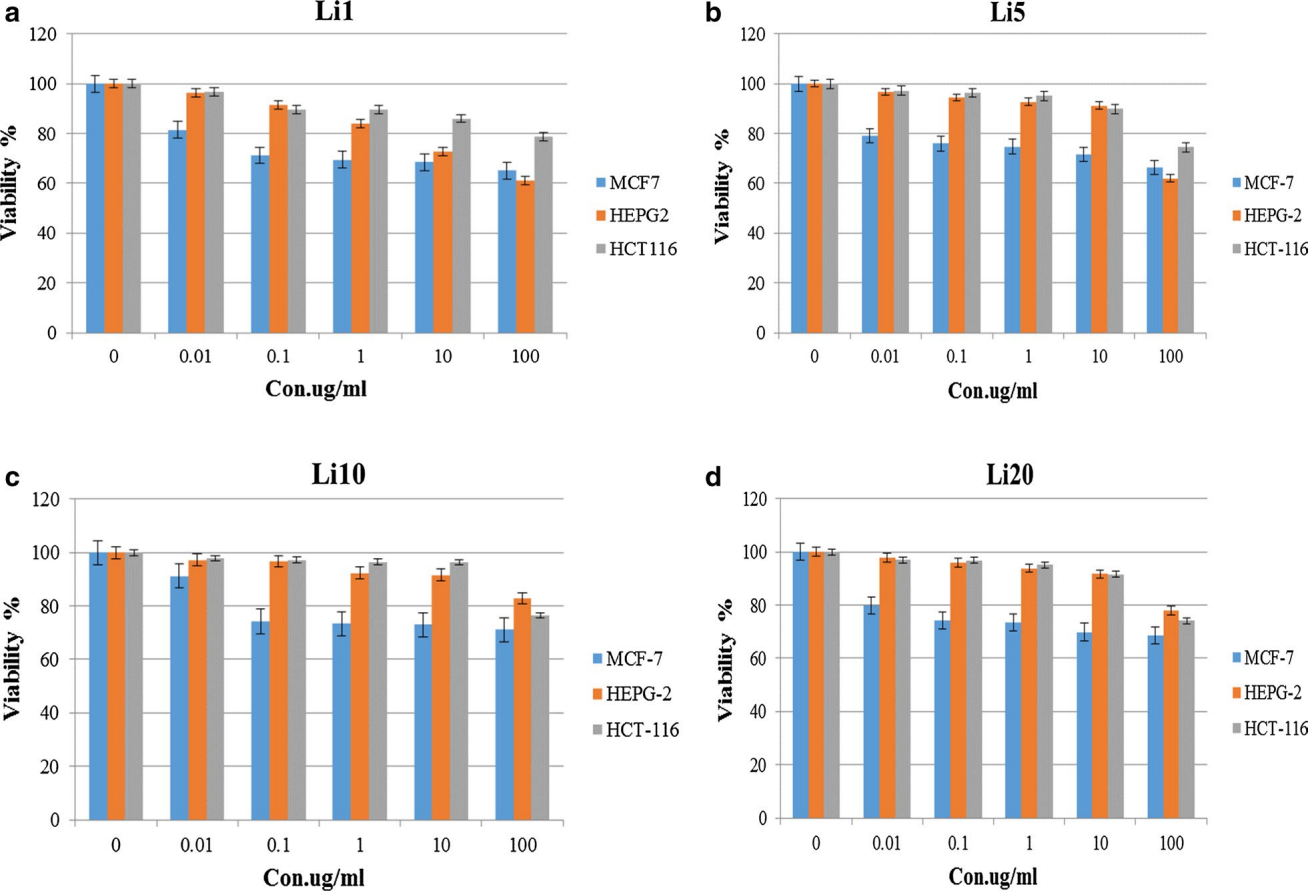
Cell viability curves of HepG2, MCF-7 and HCT-116 cancer cell lines after exposure to different concentrations of Li-TUD-1 for 72 h. Where **a** Con. 1, **b** Con. 5, **c** Con. 10 and **d** Con. 20

Moreover, the IC_50_ values on the investigated samples are listed in Table 2. The interesting point here is IC_50_ going to be increased parallel with the Li-TUD-1 concentration. While, concentration 20 exhibited different behavior where increased instead of decreased. This is—most likely—due to the presence of the extra-framework Li oxides. The calculated IC_50_ of the investigated samples are plotted in Fig. 4.

**Table 2:** The impact of TUD-1, Li and Li-TUD-1Li-TUD-1 as IC_50_ against cancer cell lines

Cell type	IC_50_		
	TUD-1	Li	Li-TUD-1/for con. (1, 5, 10, 20)
HEPG2	> 100	> 100	> 1000
MCF-7	> 100	> 100	> 1000
HCT116	> 100	> 100	> 1000

**Fig. 5:**
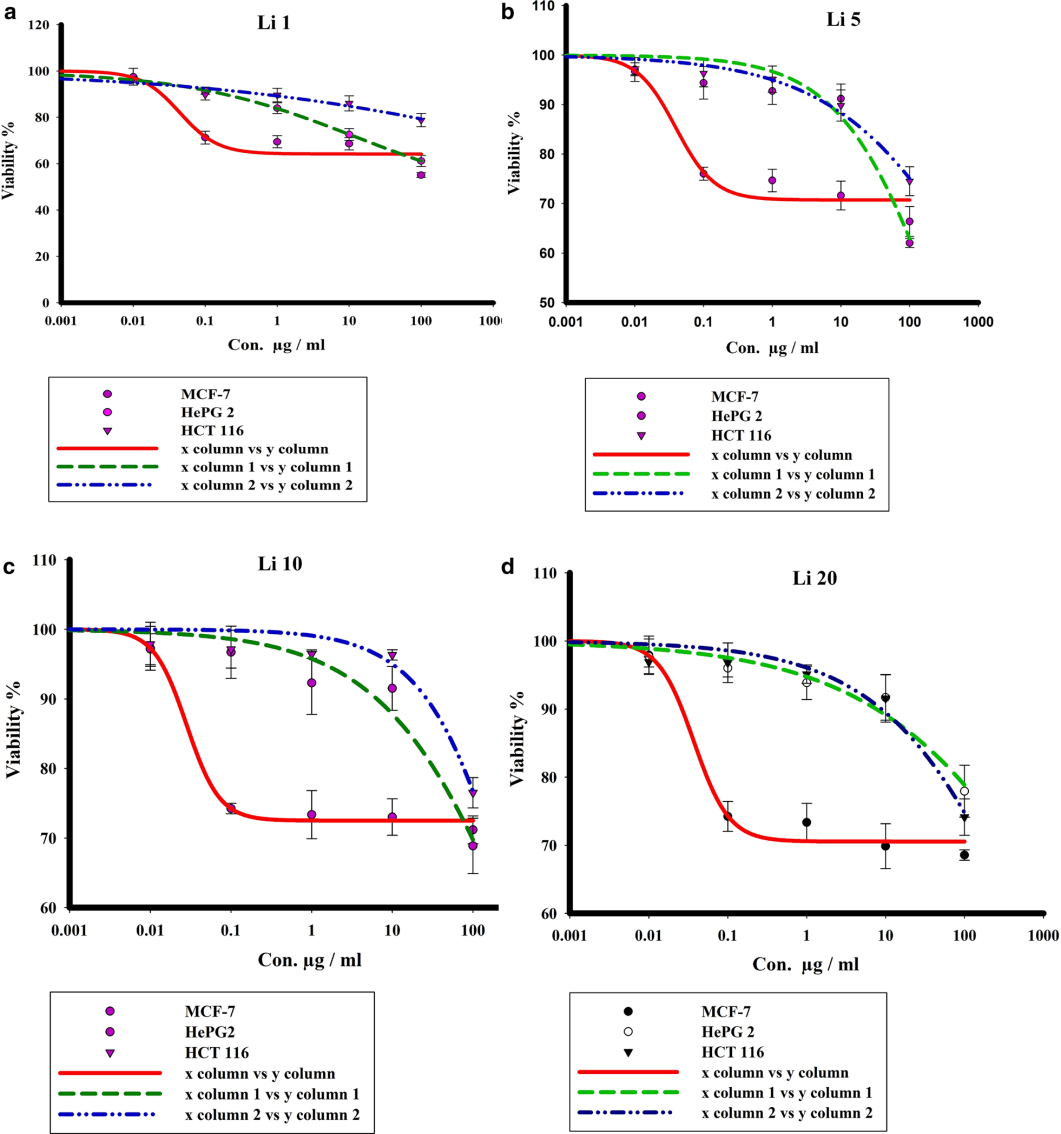
The response curves of different cancer cell lines HepG2, MCF-7 and HCT-116 after exposure to different concentrations of Li-TUD-1 for 72 h. Where **a** Con. 1, **b** Con. 5, **c** Con. 10 and **d** Con. 20

Previous results showed that there is a significant improvement in the event of the use of Li-TUD-1 (Si–Li) comparing to use elements alone. However, the difficulty lies in identifying the mechanism that caused it. Therefore, in attempt to confirm the mechanism of action, cells were stained with Acridine Orange and Ethidium Bromide (1:1) to detect apoptotic bodies, which are considered as an essential indicator of the impact mechanism (Fig. 5).

**Fig. 6:**
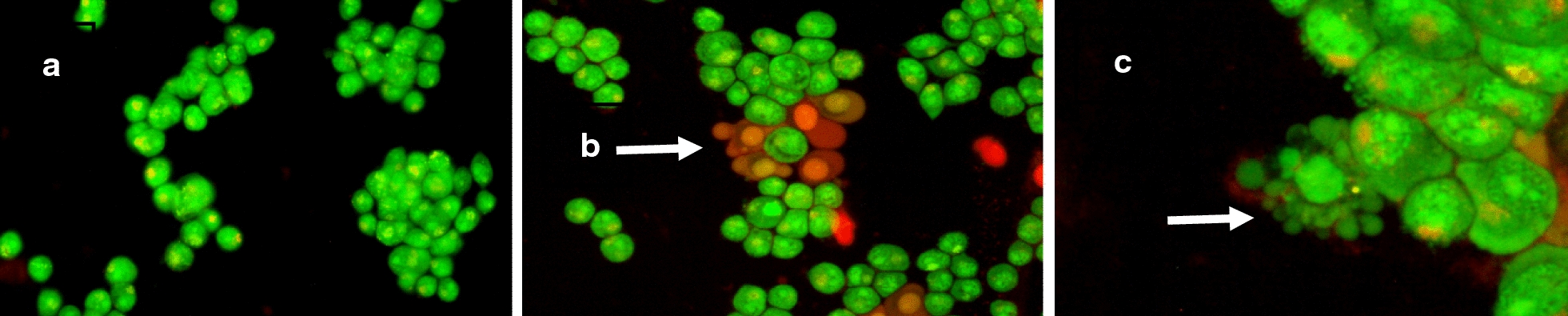
Detection of necrosis and apoptosis indicators presented on HepG-2, MCF-7 and HCT-116 cancer cell lines after treated with IC_50_ concentration of different samples where **a** control group, **b** necrotic cells, **c** apoptotic bodies presented in cells. All cells stained with (AO + EtBr), detected by Olympus fluorescent microscope after 72 h

Consequently, to explore the mechanism of action, it was necessary to survey apoptotic and necrotic indicators (apoptotic bodies, plasma membrane alteration, condensed nucleus… etc.) appear on different cancer cell lines after treated with IC_50_. On the other hand, the protective role that had been played by the Li-TUD-1 (Li-TUD-1) in preventing of necrosis and allow of apoptosis had been evaluated by count the apoptotic and the necrotic cell per 100 cell. Consequently, HepG-2, MCF-7 and HCT-116 cancer cell lines were treated with IC_50_ concentrations determined before (Fig. 6). Results showed that Li-TUD-1 has moderately decreased necrosis induction on all cancer cell lines, while the apoptotic impact was increased almost 2 times comparing to solo TUD-1 and solo Li metal (Table 3).

**Table 3:** Apoptosis induction ratio versus necrosis in different cancer cell lines per 100 cell after treated with determined IC_50_ dose of solo TUD-1, solo Li and Li-TUD-1

Cell type	Apoptosis/100 cell			Necrosis/100 cell		
	TUD-1	Li	Li-TUD-1	TUD-1	Li	Li-TUD-1
HEPG2	16	28	43	25	16	12
MCF-7	18	22	55	33	12	8
HCT116	14	25	52	30	15	10

## Discussion

Two different pathways, apoptosis and necrosis [32] control cellular death. Both of these mechanisms was acceptable as anticancer [33, 34]. However, recent studies confirmed that necrosis can be activated under many reasons like injury, infection, infraction, toxin, inflammation and the interesting cause is cancer itself [35]. In the light of these recent observations, fundamental question was present; why cancer cells choose necrosis to death instead of apoptosis. This question remained no answer until many observations confirmed that the cancer cells are trying to keep as a way as possible from what may cause the activation of the immune system [34]. For this reason, apoptosis considered the most favorite pathway to remove cancer cells under immune system control [34]. On the other hand, necrosis not only can’t activate the immune system but also leads to inflammation [35]. This challenge leads us to understand the mechanisms that can be followed in order to activate apoptosis but not necrosis. In this study, a novel molecule (Li-TUD-1) had been invented in order to induce apoptosis and inhibit necrosis at once. To achieve this goal, two molecules were combined: the first one (TUD-1) is known as a good carrier in addition to its ability to stimulate apoptosis but also causes necrosis. While the second one (Li) known for its ability to prevent necrosis. Consequently, the difference between apoptosis and necrosis in all samples before and after treated with the combined had been monitored in order to be sure that the new Li-TUD-1 had gained the requested ability [32]. Results revealed that; the lowest concentration had the lowest activity this may due to the mesoporous TUD-1 worked as a good carrier but the small Li concentration had no impact to prevent necrosis. While the moderate concentrations (5–10) had clear effect in the induction of apoptosis (Actually not significant comparing to control group) but the good point here the ability of this concentration to prevent necrosis significantly. On the other hand, the effect returned to weaken and become negligible at a concentration of 20 as—apparently—the pores of silica had been filled and no more space for more loading. This clearly demonstrates several important things like silica has a certain capacity as a carrier, and the increase in it does not increase its effect, but negatively affects the result, as the apoptosis begins disappearance gradually whereas the emergence of the necrosis. The cause of the emergence of necrosis may be due to the accumulation of excess amounts of the carrier’s capacity in the media and around cells, which prevented the oxygen absorption. Consequently, cells went to death under the induction of hypoxia but not under the impact of Li-TUD-1. The interesting point is the ability of the Li-TUD-1 to induce apoptosis and prevent necrosis in moderate concentrations. This indicates that silica as a vector is still active, and it facilitates the entry of lithium into the cells, while—at the same time—it does not affect its anti-necrosis activity [35–37]. It seems that lithium leaves the pores to begin its work in inhibiting the work of bcl2 and thus prevents or reduces the appearance of the apoptosis to a large extent. However, there are some unhealthy signs, where IC_50_ value went to be high, indicating that a part of the cells moved from necrosis to programmed cell death. Therefore, this new molecule has reduced necrosis and this is good but it did not stop the cells from dividing, which led to an increase in the proportion of cells that have not neither died, by necrosis nor programmed death.

In fact, the molecule was worked within the roadmap had been set for it significantly reducing the proportion of necrosis. But, in the opposite it appears to have reduced the cytotoxicity of TUD-1 as the cell death rate (IC_50_) has increased rather than decreased, so this new molecule has actually been prevented the occurrence of necrosis but also reduced the activity of silica catalyst for apoptosis.

## Conclusions

The goad behind the current research was to find a mechanism to reduce the necrosis while induce apoptosis in order to stimulate immune system later on. Based on the obtained results, the incorporation of Li ions in mesoporous silica matrix with different Li loading (denoted as Li-TUD-1) was successfully prepared in one-step synthesis and it showed high potential in the reduction of necrosis. However, the impact of the new Li-TUD-1 on different cancer cells remain in the beginning and further studies must be conducted in vitro as well as in vivo.
